# Fractionation of Biomolecules in *Withania coagulans* Extract for Bioreductive Nanoparticle Synthesis, Antifungal and Biofilm Activity

**DOI:** 10.3390/molecules25153478

**Published:** 2020-07-31

**Authors:** Murtaza Hasan, Ayesha Zafar, Irum Shahzadi, Fan Luo, Shahbaz Gul Hassan, Tuba Tariq, Sadaf Zehra, Tauseef Munawar, Faisal Iqbal, Xugang Shu

**Affiliations:** 1 College of Chemistry and Chemical Engineering, Zhongkai University of Agriculture and Engineering, Guangzhou 510225, China; luofan01@21cn.com; 2 Department of Biochemistry and Biotechnology (Baghdad-ul-Jadeed Campus), The Islamia University of Bahawalpur, Bahawalpur 63100, Pakistan; ayeshazafar510@gmail.com (A.Z.); s.irum66@gmail.com (I.S.); tubatariq222@gmail.com (T.T.); 3 College of Information Science and Engineering, Zhongkai University of Agriculture and Engineering, Guangzhou 510225, China; mhasan387@gmail.com; 4 Department of Botany, The Islamia University of Bahawalpur, Bahawalpur 63100, Pakistan; sadaf.zahra@iub.edu.pk; 5 Department of Physics, The Islamia University of Bahawalpur, Bahawalpur 63100, Pakistan; rana.tuseefmunawar@gmail.com (T.M.); faisal.iqbal@iub.edu.pk (F.I.)

**Keywords:** fractionation, reducing activity, biomolecules, antibiofilm, microbial infection

## Abstract

*Withania coagulans* contains a complex mixture of various bioactive compounds. In order to reduce the complexity of the plant extract to purify its phytochemical biomolecules, a novel fractionation strategy using different solvent combination ratios was applied to isolate twelve bioactive fractions. These fractions were tested for activity in the biogenic synthesis of cobalt oxide nanoparticles, biofilm and antifungal activities. The results revealed that plant extract with bioactive fractions in 30% ratio for all solvent combinations showed more potent bioreducing power, according to the observed color changes and the appearance of representative absorption peaks at 500–510 nm in the UV-visible spectra which confirm the synthesis of cobalt oxide nanoparticles (Co_3_O_4_ NPs). XRD diffraction was used to define the crystal structure, size and phase composition of the products. The fractions obtained using 90% methanol/hexane and 30% methanol/hexane showed more effectiveness against biofilm formation by *Pseudomonas aeruginosa* and *Staphylococcus aureus* so these fractions could potentially be used to treat bacterial infections. The 90% hexane/H_2_O fraction showed excellent antifungal activity against *Aspergillus niger* and *Candida albicans*, while the 70% methanol/hexane fraction showed good antifungal activity for *C. albicans*, so these fractions are potentially useful for the treatment of various fungal infections. On the whole it was concluded that fractionation based on effective combinations of methanol/hexane was useful to investigate and study bioactive compounds, and the active compounds from these fractions may be further purified and tested in various clinical trials.

## 1. Introduction

Adverse increases in the rates of microbial, fungal and viral infections worldwide prompted by compromised and human immunity are due in part to the indiscriminate use of antibiotics that enhances resistance in microbial communities against the corresponding antigens [1]. The generation of biofilms by microbes, which root in a self-produced matrix on living and non-living surfaces [2], is a peculiar behavior of microbes in inducing and producing resistance. Biofilm affinity is associated to a firm attachment of the microbe and biofilm-forming microbes have a great tendency to stick permanently to the large variety of surfaces [3]. These tiny creatures’ biofilms are protected by a layer of exopolysaccharides, which can be up to 1000 times more resistant to antimicrobials, which has increased exponentially the rate of chronic infections caused by increased resistance against the host immune system and antibiotics [4,5]. Among such microbes is *Candida albicans*, a well-known resistant nosocomial bacterium primarily known for being the main cause of infectious diseases [6] such as oral thrush [7], vaginitis [8] organ transplant recipients [9] and forms of cancer in HIV/AIDS patients. Besides resistance, the limited availability of commercial drugs effective against bacteria and the resulting toxicity has increased the global rate and effects of infections in people. This severe problem has driven the interest of researchers in developing less toxic, herbal bioactive compounds that could work against such strains of microbes. Similarly, the commonly known resistant fungus, *Aspergillus* species, responsible for pulmonary diseases, has also acquired resistance to many common drugs [10]. In order to overcome these biofilm-producers alternative treatments include the use of antibiofilm agents produced by medicinal plants as this mode of action reduces the resistance susceptibility [11]. Plants, being an enriched source of naturally occurring biologically active components, play a vital role in the prevention and treatment of diseases by boosting immunity and reducing toxicity [12,13]. Ancient plants like *W. coagulans* contain many useful bioactive molecules such as withanolide, withaferin, withacoagin [14], etc., that have been used to synthesize therapeutic drugs for the prevention and treatment of various diseases due to their reduced side effects [15,16]. *W. coagulans* belongs to the Solanaceae, a family of common traditional therapeutic plants with wide range of pharmacological applications [17], including antimicrobial, anti-inflammatory [18], antitumor [19], antihyperglycemic [20], cardiovascular, and immunosuppressive properties [21]. The constituents of *W. coagulans* include free amino acids, essential oils, steroidal lactones and esterases, widely used for their pharmacological activities [22]. A few studies have also recommended the use of withanolide, withaferin and other biological entities found in *W. coagulans* for their bioreducing potential in the synthesis of nanoparticles [23,24], and studies have reported the eco-friendly and less toxic preparation of nanoparticles and pharmacological studies using *W. coagulans* components [25].

So far, all these biological activities were tested using crude extracts containing complex mixtures of active biomolecules and the solvents-based screening, fractionation and functionalization of bioactive compounds has not been previously reported. The development of antibiofilm strategies is a major interest and also the basis of an important field of investigation that is the development of premium, environmentally friendly antibiofilm biomolecules. The present work was focused on investigate the functional role of fractions obtained using methanol and hexane with water and mixtures of methanol and hexane to purified active biomolecules from *W. coagulans* extract. For this purpose, *W. coagulans* fractions were extracted with mixtures containing different ratios of methanol and *n*-hexane and water and methanol and *n*-hexane mixtures in order to evaluate the bioactivities such as bioreducing potential for the synthesis of cobalt nanoparticles, and antioxidant, anti-biofilm and antifungal activities.

## 2. Results

**Figure 1:**
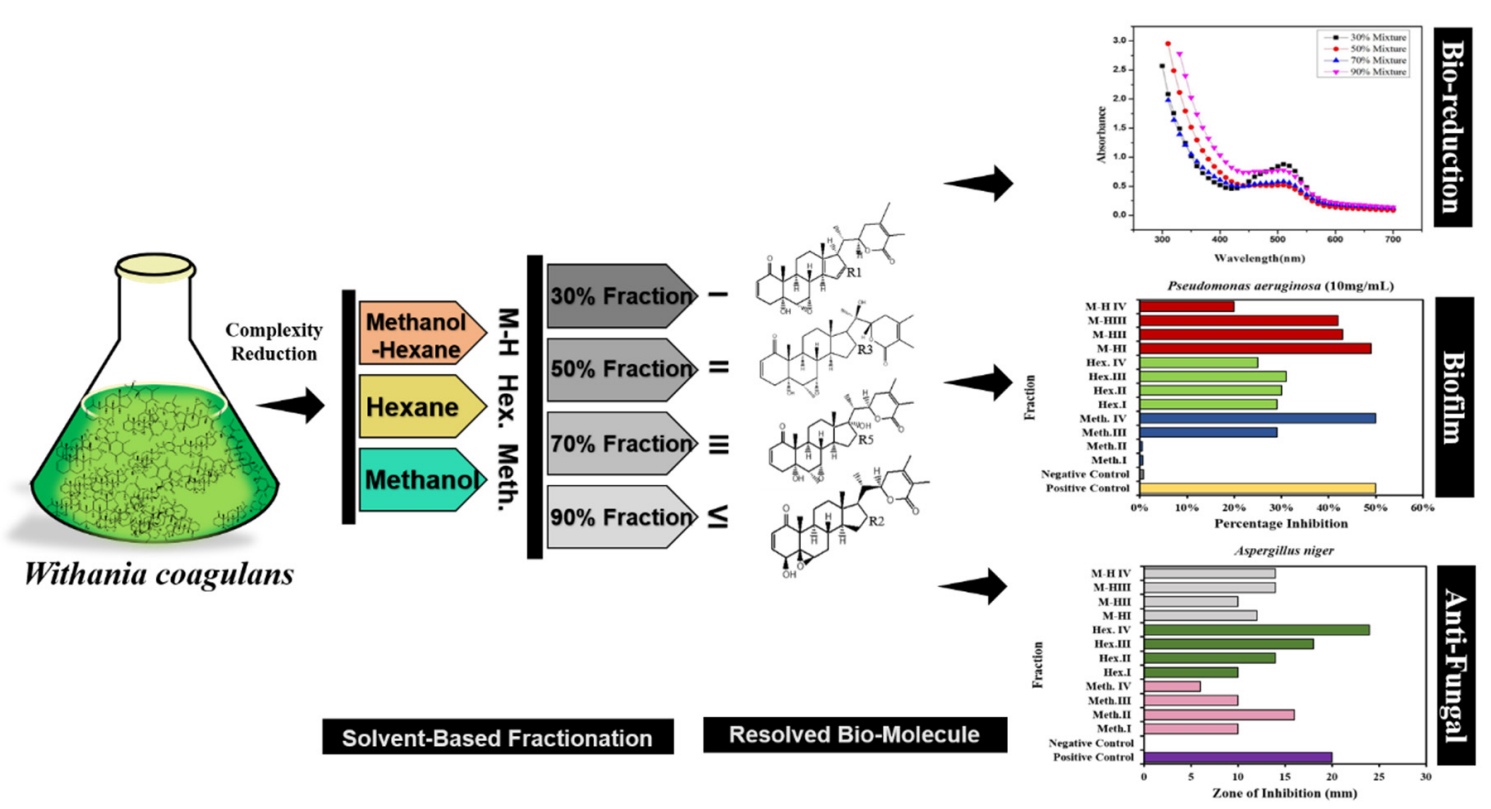
Schematic illustration of *W. coagulans* biomolecules and their applications.

The increasing resistance of microbes against antibiotics calls for the urgent discovery of unique biomolecules from extracts of plants like *W. coagulans* that are of potential interest for their antibiofilm and antifungal activity and as bioreducing agents for the synthesis of cobalt oxide nanoparticles (Co_3_O_4_ NPs). The species *W. coagulans* is highly acclaimed in the Indian ayurvedic system of medicine, where it is known for its medicinal significance in promoting physical and mental health [26,27]. Its active components include alkaloids, steroidal compounds, lactones, withaferin a [28], withanoloids [29], withanone [30], etc. that act as anti-inflammatory, anticancer, chemoprotective, hepatoprotective, immune modulatory, antifungal, antibacterial, hypocholestroemic, and radical scavenging agents [31]. The complex bioactive extract of *W. coagulans* contains potent and functional molecules that must be fractionated to simplify the complexity and provide separate bioactive molecules that can exhibit their functionalities efficiently. Different fractions of plant extract obtained using different solvents and mixtures of solvents were used to resolve the complexity of the biological entities of *W. coagulans* used as bioreducing, antibacterial, antifungal agents [32]. This fractionation route provided a means to separate, simplify and unveil the hidden active molecules in the complex. Initially using a *W. coagulans* extract, 12 different methanol, hexane and their mixture fractions in ratios of 30%, 50%, 70%, 90% were made (Figure S1) and their bio-reducing, antibiofilm and antifungal potential in vitro evaluated (Figure 1).

### 2.1. Green Synthesis of Co_3_O_4_ NPs

Pink coloured cobalt chloride solution was mixed individually with all 12 different solvent-based plant extract fractions that turned to a dark brown color upon addition and continuous magnetic stirring at 90 °C for five h. As the chemical reaction proceeded the color changed from dark brown to light brown indicating the synthesis of Co_3_O_4_ NPs (Figure S2).

#### 2.1.1. Characterization of Green Synthesized Co_3_O_4_ NPs

**Figure 2:**
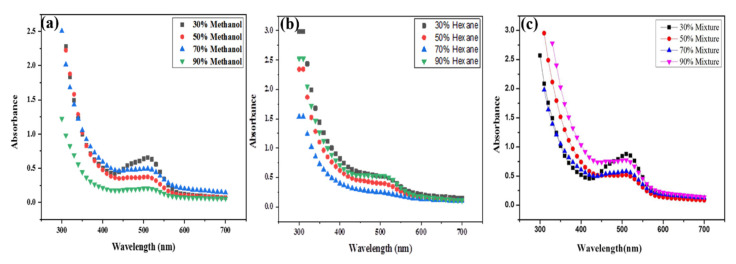
Bioreducing potential of *W. coagulans* based on: (**a**) methanol (**b**) hexane (**c**) methanol/hexane (mixture) fractions for Co_3_O_4_ NPs synthesis.

Monitoring the reduction potential of synthesized Co_3_O_4_ NPs by UV spectroscopy using the *Withania*-based fractions showed different peaks within the 500–510 nm range for different solvent fractions [33]. The methanol and water ratio results conclusively indicated that 30% methanol/H_2_O (3:1) showed the highest peak, indicating that the 30% fraction was a more active bioreducing fraction than 50% methanol/H_2_O (5:5), 70% methanol/H_2_O (7:3), or 90% methanol/H_2_O (9:1), as they all showed less bioreducing activity [34,35] (Figure 2a). Among the next four fractions based on hexane and water ratio 30% hexane/H_2_O (3:1) and 90% hexane/H_2_O (9:1) showed almost same highest peak which indicated that these fractions have more bioreducing potential than 50% hexane/H_2_O (5:5) and 70% hexane/H_2_O (7:3). Furthermore 70% hexane/H_2_O showed a much lower peak with no bio-reducing potential [36,37] (Figure 2b). Similarly, the four methanol/hexane-based fractions with different ratios (30%, 50%, 70%, 90%) were evaluated next for bioreducing potential and was indicated that 30% fraction mixture of methanol/hexane (3:1) showed a much sharper peak indicating better bioreducing potential than 50%, 70%, 90% methanol/hexane fraction mixtures [23,38] (Figure 2c).

**Figure 3:**
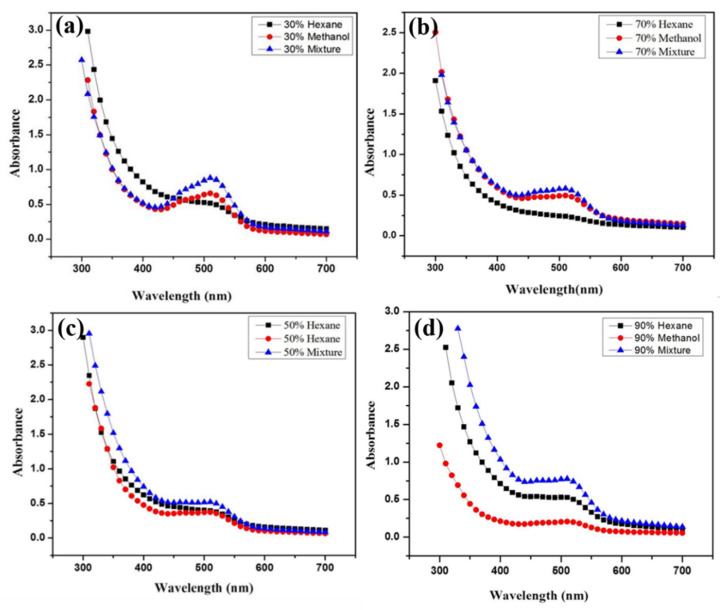
Bioreducing potential of *Withania coagulans* based on methanol, hexane and methanol/hexane (mixtures) using (**a**) 30% fraction, (**b**) 50% fraction, (**c**) 70% fraction, (**d**) 90% fraction.

For optimizing the results a comparative analysis was done between 30% fraction of methanol/H_2_O, 30% hexane/H_2_O and 30% methanol/hexane and the results demonstrated that out of all mixtures the 30% methanol/hexane (3:7) fraction mixture showed a much sharper peak. meaning it had a higher bioreducing ability than 30% methanol/H_2_O and 30% hexane/H_2_O fraction, as seen in Figure 3a. 

Among the 50% fractions, 50% methanol/hexane (5:5) fraction mixture showed higher peaks corresponding to a higher bioreducing potential than 50% methanol/H_2_O and 50% hexane/H_2_O, but 50% hexane/H2O and 50% methanol/H_2_O showed almost the same peak and almost the same bioreducing potential (Figure 3b).

Next, among the different 70% fractions of *W. coagulans*, the 70% methanol/hexane (7:3) mixture fraction showed a high peak with higher bioreducing potential than 70% methanol/H_2_O and 70% hexane/H_2_O. Here 70% methanol/H_2_O showed a much sharper peak (indicating better bioreducing potential) than the 70% hexane/H_2_O fraction, as illustrated in Figure 3c. Finally, out of all the 90% fractions of *W. coagulans*, 90% methanol/hexane (9:1) fraction mixture showed the highest peak indicating a higher bioreducing potential than 90% methanol/H_2_O and 90% hexane/H_2_O. Here different results were observed because 90% hexane/H_2_O shows a much sharper peak than 70% methanol/H_2_O meaning that 70% hexane/H_2_O fraction has higher bioreducing ability than 70% methanol/H_2_O (Figure 3d).

#### 2.1.2. XRD Analysis of Co_3_O_4_ NPs

**Figure 4:**
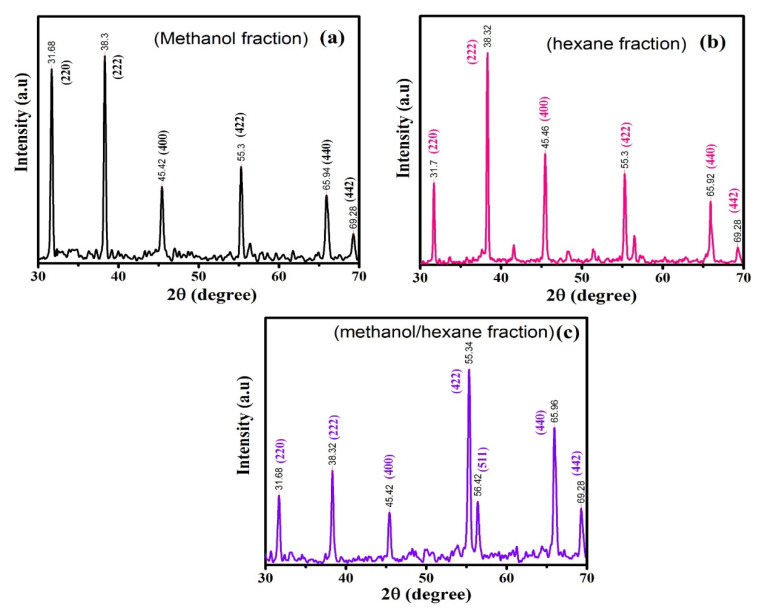
XRD analysis of Co_3_O_4_ nanoparticles based on solvent fractions: (**a**) methanol fraction (**b**) hexane fraction, (**c**) methanol/hexane fraction.

XRD diffraction was used to define the crystal structure and phase composition of the produced NPs. The XRD patterns of the samples obtained with different solvent fraction ratios are presented in Figure 4a–c. The observable diffraction pattern of materials obtained using methanol (fraction (a)), hexane (fraction (b)) and methanol/hexane (fraction (c)) were well-matched with Co_3_O_4_. The diffraction patterns of the methanol fraction were thus consistent with JCPDS Card No. 01-080-1534, hexane fraction (b) with JCPDS Card No. 01-074-1657, and methanol/hexane fraction (c) with JCPDS Card No. 01-076-1802, respectively. The peaks and related planes are indicated in Figure 4. The XRD results show that none of the samples have any characteristic peaks due to impurities, which shows that the grown samples have outstanding crystalline nature. The lattice parameters (*a*) and unit cell volume (*v*) of the samples were calculated using the following formula:

(1) $$\frac{1}{d^{2}}=\frac{h^{2}+k^{2}+l^{2}}{a^{2}}$$

(2) $$v=a^{3}$$

Where (hkl) are the miller index, ‘*d*’ is d-spacing, and ‘*a*’ is lattice constant. The calculated values are listed in Table 1. The average crystallite size (*D*) of all synthesized samples was determined by using the well-known Debye–Scherer Formula [39,40]:

(3) $$D=\frac{K\lambda }{\beta \cos\theta }$$

In these equations *K* is the shape factor having value (0.94), λ is the wavelength of X-ray (1.5406 Å), *β* is the full width at half maxima. From the results, it can be concluded that the crystallite size follows the trend b (59 nm) > a (50 nm) > c (49 nm) (Table 1). The dislocation density (*δ*) and d-spacing can be calculated by:

(4) *δ* = 1/*D*^2^

(5) 2*dsin θ = n λ*

where ‘*λ*’ is the wavelength of X-rays in Å, ‘*θ*’ is the diffraction angle (Bragg angle) in degrees, *n* is the order of diffraction which is the spacing between adjacent crystal planes. The calculated values are listed in Table 1. The results show that d-spacing varies directly with crystallite size while dislocation density varies as square inverse of crystallite size.

**Figure 5:**
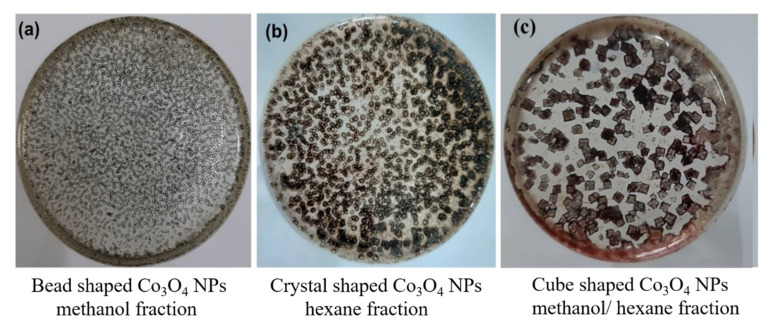
Variation in Co_3_O_4_ nanoparticle shape based on solvent fractions: (**a**) methanol fraction (**b**) hexane fraction, (**c**) methanol/hexane fraction.

Furthermore, compound microscopy results (Figure 5a–c) show that changing the nature of the solvent influenced on the shape of Co_3_O_4_ NPs. Figure 5a shows bead-shaped Co_3_O_4_ NPs obtained using methanol solvent extract as reducing agent [41] while in Figure 5b the shape of Co_3_O_4_ NPs obtained with hexane was different because of the different biomolecules present as compared to methanol solvent [42]. In the case of a mixed ratio of methanol and hexane solvents (Figure 5c), the Co_3_O_4_ NPs were cube-shaped, most probably because of the action of different active biomolecules in this fraction when they reduce the cobalt nanoparticles [27,43].

From the above results, we can conclude that the active biomolecules exhibiting reducing potential found in the methanol/hexane fraction were proven to have the best bioreducing potential in the synthesis of Co_3_O_4_ NPs. Among the different fractional concentrations of similar solvents 30% fraction showed the best bioreducing efficiency. This means that when preparing fractions with these three solvents, and running a separate solvent fraction-based reaction, the 30% fraction will provide more significant results as previously reported [44,45]. It shows a well-defined sharp peak for every solvent containing a 30% solvent faction. The exposed binding sites for the binding of cobalt precursors and saturating the metal by biochemical agents in order to provide stability was done by solvent-based fractionation of *Withania* extract as reported earlier [46].

The scheme (Figure 1) shows a double dip strategy where the nature and concentration of a solvent reduce the complexity, provide active sites and finally highlight the functional activity of the biomolecules. This solvent fractionation actually works similarly to an enzyme substrate reaction, as active sites are provided as product gets generated. Here the fractionation helps expose and present the active sites by reducing the complexity and generating Co_3_O_4_ NPs. In the next level of optimization, the concentration was kept constant and the solvent was altered. The results showed that the mixture of methanol/hexane was a hybrid solvent that reinforced the characteristic properties of each solvents. Conclusively in order to optimize our study, mixtures of methanol/hexane, at all concentrations provide the best reduction capacity. Thus, to reduce complexity, unlocking the bioactive molecules in methanol/hexane mixtures of 30% fraction should provide an excellent lead for identifying compounds good at reducing cobalt to Co_3_O_4_ NPs.

### 2.2. Biofilm Activity of Prepared W. coagulans Fractions

Bioactive fractions from *W. coagulans* (12 different fractions) were evaluated for antibiofilm activity against the drug sensitive strains *Pseudomonas aeruginosa* and *Staphylococcus aureus* in 96 well micro-titer plates. The purpose was to evaluate the potential of the 12 different fractions to inhibit the growth of a preformed biofilm already established in the wells of the micro-titer plate [47]. In anti-biofilm assay biofilm was induced to grow on 96 well micro-titer plates by adding 100 µL nutrient broth, 100 µL plant extract and 20,100 µL bacterial culture in each well and incubating for 24 h at 37 °C then staining the next day with crystal violet (dye) give a dark blue color to the well where biofilm formation took place (Figure S3). Crystal violet is a dye that binds non-specifically to negatively charged surface molecules such as the polysaccharide matrix of biofilms and stains them with a blue color so it is generally used to estimate biofilm biomass [48], so a reduction in blue color indicates biofilm inhibition by different tested plant fractions.

### 2.3. Antibiotic Selectivity

**Figure 6:**
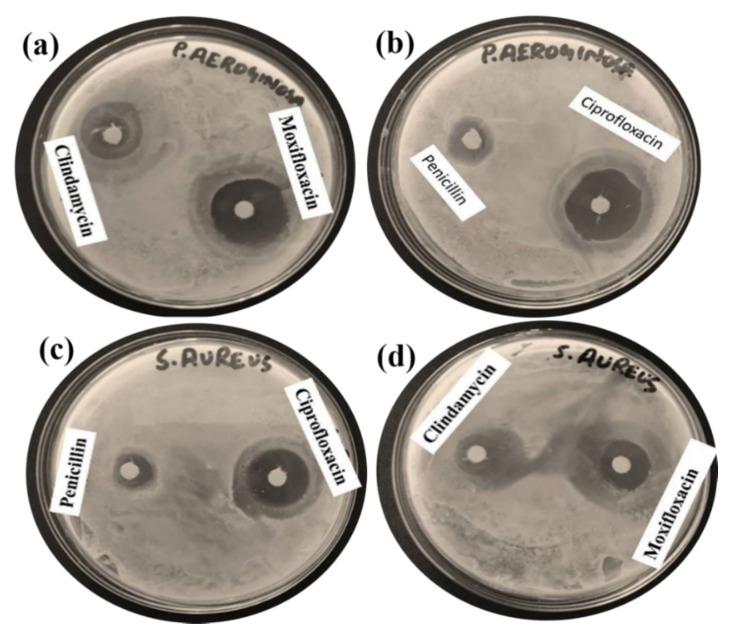
Antibiotic selectivity of (**a**) clindamycin, moxifloxacin (**b**) penicillin, ciprofloxacin against *P. aeruginosa* (**c**) penicillin, ciprofloxacin (**d**) clindamycin, moxifloxacin against *S. aureus.*

First an effective positive control for *P. aeruginosa* and *S. aureus* (drug sensitive strains) was established by treating with four different antibiotics (clindamycin, moxifloxacin, penicillin and ciprofloxacin). The results showed that moxifloxacin and ciprofloxacin were more active drugs against the *P. aeruginosa* strain as indicated by a larger zone of inhibition shown by the drugs (Figure 6a,b) but ciprofloxacin was a more effective antibiotic against *S. aureus* as shown by its larger inhibition zone (Figure 6c). Thus, the strong antibiotic ciprofloxacin was selected to test the *W. coagulans*-based 12 different fractions of methanol and hexane and their mixtures to evaluate the biofilm inhibition potential against *P. aeruginosa* and *S. aureus* at concentrations of 5 mg/mL and 100 mg/mL.

#### 2.3.1. Biofilm Inhibition Potential of *W. coagulans* Fraction against *P. aeruginosa*

Ciprofloxacin, being a positive control against *P. aeruginosa*, shows a reduction of dark blue color of the dye (crystal violet) in the first well and solvent blank without bacterial strain marked as first negative control that does not contain bacteria so no biofilm formation occurred there, thus no crystal violet dye staining was observed (Figure S3), leaving a colorless well indicating the absence of bio-film formation. As a second negative control a well was loaded with 55 mg/mL of *P. aeruginosa* without the plant extract and blue colored biofilm was observed. Color reduction of the dark blue dye in the micro-titer plate well gave a rapid qualitative analysis of biofilm inhibition potential by the crystal violet staining technique that was measured as a percentage inhibition of biofilm formation. With the positive control, ciprofloxacin, the percentage inhibition against *P. aeruginosa* was found to be 50%, and it was 0.7% with the negative control. 

After running the successful controls, the *Withania*-derived solvent-based fractions were assessed. For 30% methanol (Meth.^I^) the inhibition was 0.6%, for 50% methanol (Meth.^II^) it was 0.5%, for 70% methanol (Meth.^III^) it was 29% and for 90% methanol (Meth.^IV^) the inhibition reached 50%. Hexane was next and 30% hexane (Hex.^I^) exhibited 29% inhibition, 50% hexane (Hex.^II^) showed 30% inhibition, 70% hexane (Hex.^III^) showed 31% and 90% hexane (Hex.^IV^) gave about 24% inhibition.

**Figure 7:**
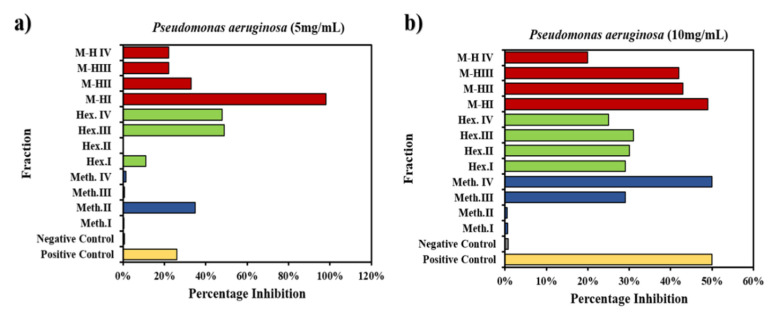
Biofilm activity of *W. coagulans* 12 fraction against *P. aeruginosa* with concentration (**a**) 5 mg/mL (**b**) 10 mg/mL.

The third series includes mixtures of methanol and hexane, among which 30% methanol-hexane (M−H^I^) showed 49% inhibition, 50% methanol-hexane (M−H^II^) 43%, 70% methanol-hexane (M−H^III^) 42% and 90% methanol-hexane (M−H^IV^) showed only 20% inhibition of biofilm formation. Overall Meth.^IV^ exhibited a 100% percentage inhibition of biofilm production with respect to control. On average Meth. inhibited 40%, Hex. inhibited 57% and methanol-hexane mixture inhibited 77% with respect to control. Hence the solvent mixture super-combination showed superior results on average at all concentrations by decoding the complexity with the hybrid mixture of solvents. Biofilm formation by dye degradation and calculated inhibitions are shown in Figure 7a. These results are relevant to previous work done using plant extracts against the biofilm activity [49].

Similarly, when using the 10 mg/mL extract against *P. aeruginosa* where the positive control showed 26% inhibition of biofilm and 0.7% of inhibition for the negative control, 0.5% > 35% > 0.6%> 1.5% inhibition was seen for Meth.^I^ > Meth.^II^ > Meth.^III^ > Meth. ^IV^. Moving to the next solvent fraction Hex.^I^ > Hex.^II^ > Hex.^III^ > Hex.^IV^ (11% > 0.3%> 49%> 48%) and lastly, for the mixture fraction M−H^I^ > M−H^II^ > M−H^III^ > M−H^IV^ (98% >33% > 22%) as depicted in Figure 6b along with biofilm formation (Figure S4). The best fractions M−H^I^, Hex.^III^, Hex.^IV^, Meth.^II^ and M−H^II^ provided an outstanding inhibition representing 277%, 88%, 85%, 34% and 26% more than the control. As a result, Meth provided 36% inhibition with respect to control, Hex exhibited 4% more inhibition with respect to control whereas the excelling M−H mixture exhibited 68% more inhibition with respect to the control on average. Some fractions had previously shown significant inhibition with 5 mg/mL *Withania* solution against *P. aeruginosa* [50] but changing the concentration to 100 mg/mL the bio-film percentage inhibition increased even above the control level, showing higher antibacterial activity as shown in Figure 7b.

#### 2.3.2. Biofilm Inhibition Potential of *W. coagulans* Fractions against *S. aureus*

The activity of concentrations of each fraction up to 10 mg/mL against *S. aureus* was observed. Biofilm formation against *S. aureus* strain was done with ciprofloxacin as positive control which was found to be active against the drug sensitive *S. aureus* strain as shown by the white colour of wells. A negative control was also added (Figure S5). 

**Figure 8:**
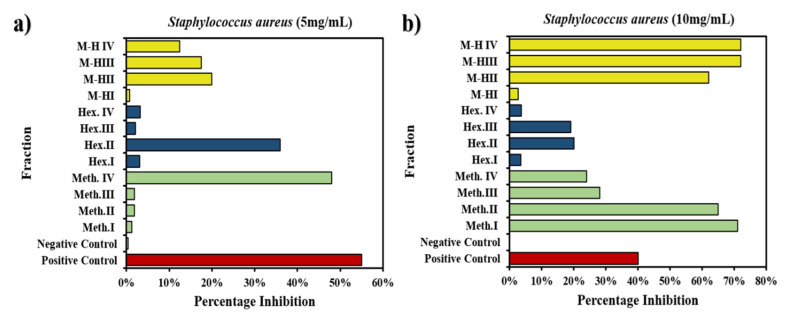
Biofilm activity of *W. coagulans* 12 fraction against *S. aureus* with concentration (**a**) 5 mg/mL (**b**) 10 mg/mL.

The controls gave 55% and 0.4% inhibition, respectively. For the other 12 fractions a concentration of 55 g/mL was used that provided no significant or results as shown in Figure 8a where the positive control inhibition was 55% and that of the negative control was 0.4%. Meth.^I^ > Meth.^II^ > Meth.^III^ > Meth.^IV^ values were 1.2% > 1.8% > 1.9% > 48%. For hexane, i.e., Hex.^I^ > Hex.^II^ > Hex.^III^ > Hex.^IV^ the inhibition was 3.1% > 36% > 2.1% > 3.2% and for mixtures M−H^I^ > M−H^II^ > M−H^III^ > M−H^IV^, percentage inhibitions of 0.8% > 20% > 17.5% > 12.5% were exhibited which were quite insignificant against such a resistant strain and at a such minute concentration.

Next the change in concentration up to 10 mg/mL against *S. aureus* showed significant results, whereby the positive control showed 40% inhibition and the negative one showed 0% inhibition. In the first fraction series Meth.^I^ > Meth.^II^ > Meth.^III^ > Meth.^IV^ the inhibition was 71% > 65% > 28% > 24%. For hexane fractions, i.e., Hex.^I^ > Hex.^II^ > Hex.^III^ > Hex.^IV^ the results showed 3.5% > 20% > 19% > 3.7% inhibition and the percentage inhibition was calculated as 2.7% > 62%> 72% > 72% for M−H^I^ > M−H^II^ > M−H^III^ > M−H^IV^ as shown in Figure 8b. Compared to the control M−H^III^ >M−H^IV^ > Meth.^I^ > Meth.^II^ > M−H^II^ exhibited 80% > 80% > 78% > 62% > 55% more biofilm formation indicating an outstanding result at the particular dilutions that revealed the presence of antibacterial biomolecules in such fractions. On average Meth. showed 18% more inhibition, Hex. showed only 28% inhibition with respect to control and M−H was superior, exhibiting more film formation with 30% inhibition. The present observations regarding bacterial biofilm formation match the work rewported by previous researchers [51].

### 2.4. Antifungal Activity of Prepared W. coagulans Fractions

#### 2.4.1. Antifungal Activity of Prepared *W. coagulans* against *A. Niger*

**Figure 9:**
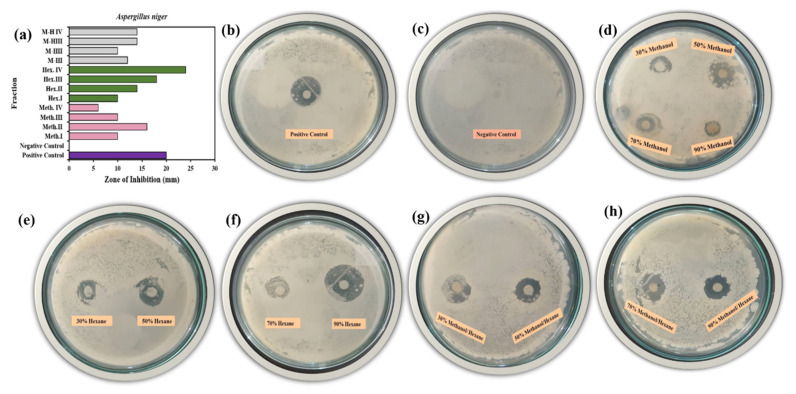
Antifungal activity of 12 *W. coagulans* fractions against *A. niger* measured by zone of inhibition: (**a**) Positive control; (**b**) negative control; (**c**) methanol fractions; (**d**) hexane fraction 30%, 50%; (**e**) hexane fraction 70%, 90%; (**f**) methanol/hexane fraction 30%, 50% (**g**); methanol/hexane fraction 70%, 90% (**h**).

The antifungal activity was evaluated using all 12 different fractions extracts of methanol and hexane and their mixtures using plant extract of *W. coagulans* against *A. niger* and *C. albicans* by the disc method [52]. The active biomolecules were resolved into simple *W. coagulans* plant molecules that exhibited antifungal activity. The active principal molecules were measure and made visible by the zone of inhibition produced by the fraction molecules against the specific strains (Figure 9a–h).

The tested fractions provided significant results as follows: amphotericin B at concentration (10 mg/mL) was used as standard for both fungus strains that were pathogenic [53]. The positive control shows good antifungal activity against *A. niger* as indicated by the large clear inhibition zone (20 mm) whereas the negative control exhibited no clear zone of inhibition as shown in Figure 9b,c. The tested concentrations beginning with Meth.^I^ > Meth.^II^ > Meth.^III^ > Meth.^IV^ exhibited 10 mm > 16 mm > 10 mm > 6 mm inhibition, with an average of 50% antifungal activity compared to the control (Figure 9d). Next is the hexane fractions, Hex.^I^ > Hex.^II^ > Hex.^III^ > Hex.^IV^ showing 10 mm >14 mm >18 mm > 24 mm zone of inhibition with 83% agreement with the control (Figure 9e,f). Finally M−H^I^ > M−H^II^ > M−H^III^ > M−H^IV^ where the zone of inhibition provided 62% similar result on average with control and 12 mm > 10 mm > 14 mm > 14 mm inhibition zones, respectively (Figure 9g,h). Surprisingly Hex.^IV^ showed 20% more antifungal activity than the control against *A. niger*.

#### 2.4.2. Antifungal Activity of Prepared *W. coagulans* against *C. albicans*

**Figure 10:**
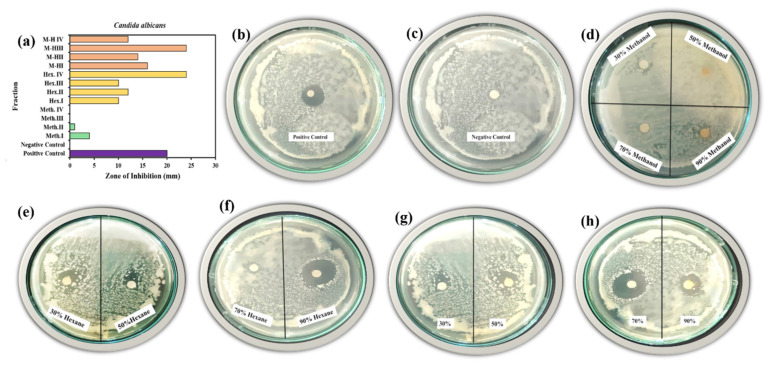
Antifungal activity of 12 *W. coagulans* fractions against *C. albicans* (zone of inhibition): (**a**) Positive control (**b**) negative control (**c**) methanol fractions (**d**) hexane fraction 30%, 50% (**e**) hexane fraction 70%, 90% (**f**) methanol/hexane fraction 30%, 50% (**g**) methanol/hexane fraction 70%, 90% (**h**).

*C. albicans* showed a 20 mm zone of inhibition with the positive control amphotericin B, an effective drug against this strain (Figure 10a–h). The negative control provided no zone of inhibition indicating no antifungal activity (Figure 10c). On further treatment the 12 fractions provided significant results, where Meth. provided 6% antifungal activity, Hex. 70% activity and M−H 83% activity with respect to the control.

Individually Meth.^I^ > Meth.^II^ > Meth.^III^ > Meth.^IV^ provided 4 mm > 1 mm > 0 mm > 0 mm of inhibition zone (Figure 10d), Hex.^I^ > Hex.^II^ > Hex.^III^ > Hex.^IV^ had 10 mm > 12mm > 10 mm > 24 mm inhibition (Figure 10e,f). Finally 16 mm > 14 mm > 24 mm > 12 mm zones of inhibition were measured for M−H^I^ > M−H^II^ > M−H^III^ > M−H^IV^ fractions (Figure 10g,h). With *C. albicans* Hex.^IV^ and M−H^III^ exhibited 20% more antifungal activity that the control.

The antibacterial and antifungal activity using *W. coagulans* was proven to be significant because of the biomolecules initially present in complex form that were resolved into simple and more functionally active groups by the solvent-based fractionation method. Owing to such a strategy and the significant activity this set of optimizations can be incorporated in the medicinal field in order to combat bacterial and fungal infections. Plant extracts have shown a variety of potentials such as reducing, antioxidant, synthetic, and medicinal activities due to the presence of numerous bio-molecules that exist in different parts of the plant. Depending upon the nature each show different extents of variation in their capabilities due to the presence of some additional biomolecules and the varying concentrations of those biomolecules. Considering *Withani*, it is truly rich in phenols, flavonoids, alkaloids, steroids and other complex structures that provide reducing, antibacterial and antifungal activities. The results of this work show that the separation of these components using different solvents such as water, hexane, methanol, acetone, etc., enhanced the activities by aiding in resolving the complexity, dissolving components of different nature according to their solubility in different solvents, combining the biomolecules for effective interaction and thus showing their potentials at their maximum level. Similarly, *Withania* had shown antibacterial activity against *Salmonella typhi, Klebsiella pneumonia, S. aureus* with percentage antibacterial activities as 43%, 0%, 73% respectively. *Withania*-decorated iron rods enhanced the activity up to 30% for *S. aureus* and *P. aeruginosa,* whereas *Withania* showed less inhibition against a *Brucella* strain. Multiple examples have shown that the bacterial inhibition of crude extracts was not so high as that achieved by using solvent- based fractionation methods that enhance the values and activity to a significant level. Antifungal activity was exhibited against various strains such as *A. flavus*, *A. niger, Penicillium* and *Alternaria alternate*, where a significant 6–10 mm zone was measured. Thus, the addition of solvents, mixtures of solvents, and the concentration help in simplifying the complex structure of the plant extracts that displayed much higher activities, including bioreduction, antibacterial and antifungal properties.

## 3. Materials and Methods

### 3.1. Plant Material

Plant of *W. coagulans* was obtained from a local market in Bahawalpur, Pakistan in September 2018. Fresh plant was washed three time with distilled H_2_O and kept in the shade until it was completely dried, then it was crushed into powder form for further use.

### 3.2. Preparation of Plant Extract

Whole plant was dried and crushed using a pestle and mortar to obtain a fine powder, then 10 g of extract powder was dipped in different concentrations of methanol and hexane to make 12 different fractions with ratios of 90%, 70%, 50%, 30% (final volume 200 mL). After overnight incubation the extracts were filtered and the filtrates were dried in an incubator at 37 °C. These powder extracts then used to check the bioreducing, antifungal and biofilm activities.

### 3.3. Synthesis of Cobalt Oxide Nanoparticles

For the synthesis of cobalt oxide nanoparticles, a 0.5 M solution of cobalt chloride was prepared Flasks containing 40 mL cobalt chloride solution and 10 mL plant extract (90%, 70%, 50%, 30%) were prepared, put on a magnetic stirrer (150 rpm) and kept there for 4 h at 90 °C as a reaction occurred indicated by a change in color confirming the synthesis of nanoparticles. After this the mixture was centrifuged at 6000 rpm for 10 min., the pellet was separated and dried for characterization.

### 3.4. UV-Vis Spectroscopy

All 12 fractions were subjected to UV-Vis spectroscopy (Instrument model VT05404-0998, Biotek, Winooski, VT, USA) at predetermined time intervals to confirm the formation of cobalt nanoparticles and the wavelength was noted. Peaks between 550–510 nm give a positive indication of nanoparticle synthesis. Also, the color changes of reaction mixtures were used as evidence of cobalt oxide nanoparticle formation

### 3.5. Morphology Analysis of via Compound Microscope

The dried form of the cobalt oxide nanoparticles was uniformly distributed in Petri plates with relevant solvent and allowed to dry. Later compound microscopy (model IM-850, IRMECO GmbH, Hamburg, Germany) was used to observe the morphological variations in all three fractions.

### 3.6. Biofilm Assay of W. coagulans Fraction

Biofilm assays were performed by a crystal violet staining assay. The effect of extracts on biofilm formation was evaluated in 96-well polystyrene plates. Firstly, the 96-well micro-titer plates were washed with sterile distilled water, air dried and then oven-dried at 60 °C for 45 min. Briefly, nutrient broth, standard drug (ciprofloxacin) and bacterial culture were used as positive control while nutrient broth, distilled water and bacterial culture were used as negative control. Nutrient broth, plant fractions and bacterial culture were added to each micro-plate and incubated at 37 °C for 24 h. After that staining with 0.1% crystal violet was performed and the OD was recorded at 630 nm using an ELISA reader (model IM-850, IRMECO GmbH, Hamburg, Germany) and % inhibition was calculated by following formula:

(6) % inhibition = (A_0_ − A_1_)/A_0_ × 100

where A_0_ is absorbance of negative control and A_1_ is the absorbance of the plant fractions

### 3.7. Antifungal Activity of W. coagulans Fraction

Fresh plant was washed two times with distilled water and allowed to dry at room temperature for 3 to 4 days. The dried material was ground and extracted separately by making different methanol and hexane fractions. The extracts were filtered and the filtrate was dried. All extracts fractions were stored at 4 °C and used for the bioassays. The plant extracts were tested against two important fungal pathogens, *C. albicans* and *A. niger,* obtained from the laboratory of the Department of Biochemistry and Biotechnology (Islamia University Bahawalpur). All cultures were maintained on SDA agar at 37 °C. Overnight cultures on SGA slants at 37 °C were used to prepare the fungal inoculum to be used in the antimicrobial assays. The antifungal activity of *W. coagulans* methanolic and hexane extracts was measured according to the disc diffusion method. Sterile blank discs of 6 mm diameter were soaked with the prepared *W. coagulans* extracts to give a final concentration of 10 mg/mL, respectively. The discs were then placed firmly on a SDA surface which has been previously seeded with *C. albicans* strain suspension. The same steps were repeated for the A. *niger* strain. All plates were incubated overnight at 37 °C. Throughout this experiment, a blank disc impregnated with sterile distilled water represented as negative control while a disc soaked with 100 μL of amphotericin B was the positive control. The susceptibility of each *Candida* spp. was determined by the diameter of the growth inhibited zone surrounding the disc.

## 4. Conclusions

Twelve different *W. coagulans*-based fractions prepared using combinations of different solvents (methanol, hexane) and their mixture were used to study the effect of different solvent combinations on various biological activities. Plant fractions of different concentration (30%, 50%, 70%, 90%) were used. These fraction were used to investigate the bioreducing potential of the plant extracts containing complex biomolecule mixtures, it was found that collectively 30% fraction of methanol. hexane, and mixture of methanol-hexane provided the highest reducing potential for the synthesis of cobalt oxide nanoparticles. Results also showed that 90% methanol/hexane and 30% methanol/hexane were more active against biofilm formation of *P. aeruginosa* and *S. aureus* so these fractions could be used for treatment of various drug resistance-related bacterial infections. A 90% fraction of hexane/H_2_O showed excellent antifungal activity against *P. niger* and *C. albicans*, while 70% methanol/hexane show good antifungal activity for *C. albicans,* so these fractions are potentially useful for the treatment of various fungal infections. This solvent-based fractionation method provides a direct means to reduce the complexity of the *W. coagulans* extracts and reveal the strong bioreducing, antifungal and antibiofilm activities and optimize the particular activity for practical applications. This provides a cost-effective, ecofriendly, non-toxic and effective source for medicinal and synthetic applications.

## Figures and Tables

**Table 1:** Structural parameters of grown samples.

Samples	a(Å)	b(Å)	c(Å)	d-Spacing	Volume (Å^3^)	Crystallite Size (nm)	Dislocation Density δ (nm)^−2^ × 10^−4^
**A**	8.04702	-	-	1.8365	521.0805	50	4.000
**B**	8.07016	-	-	1.9657	525.5884	59	2.870
**C**	8.06895	-	-	1.8362	525.3527	49	4.160

## Supplementary Materials

The following are available online, Figure S1 Screening strategy for exploring bioactive fraction of *W. coagulans*; Table S1 Different solvent fractionation of *Withania coagulans*.
