# Supplementary material for: Fractionation of Biomolecules in Withania coagulans Extract for Bioreductive Nanoparticle Synthesis, Antifungal and Biofilm Activity
Source: Molecules. 2020 Jul 31;25(15):3478. doi: 10.3390/molecules25153478 (PMC7435783; doi:10.3390/molecules25153478)
Supplement: Supplementary file 1 [file molecules-25-03478-s001.pdf]

Fractionation of biomolecules in *Withania coagulans* extract for bioreducing in nanoparticles synthesis, antifungal and biofilm activity

Murtaza Hasan[1,2] ǀ *, Ayesha Zafar[2] ǀ , Irum Shahzadi[2], Fan Luo[1], Shahbaz Gul Hassan[3], Tuba Tariq[2], Sadaf Zehra[4], Tauseef Munawar[5], Faisal Iqbal [5], Xugang Shu[1]*

[1]College of Chemistry and Chemical Engineering, Zhongkai University of Agriculture and Engineering, Guangzhou 510225, China

[2]Department of Biochemistry and Biotechnology (Baghdad-ul-Jadeed Campus), The Islamia University of Bahawalpur, 63100, Pakistan

[3]College of Information Science and Engineering, Zhongkai University of Agriculture and Engineering, Guangzhou, China

[4]Department of Botany, The Islamia University of Bahawalpur, Bahawalpur 63100, Pakistan

[5]Department of Physics, The Islamia University of Bahawalpur, Bahawalpur 63100, Pakistan.

*Correspondence: murtaza@zhku.edu.cn xgshu@21cn.com Tel.: 86-020-8900-3114

ǀ These authors contributed equally to this work

***Corresponding authors**

Murtaza Hasan **murtaza@zhku.edu.cn**

Xugang Shu **xgshu@21cn.com**

‖These authors contributed equally to this work.

Experimental Section

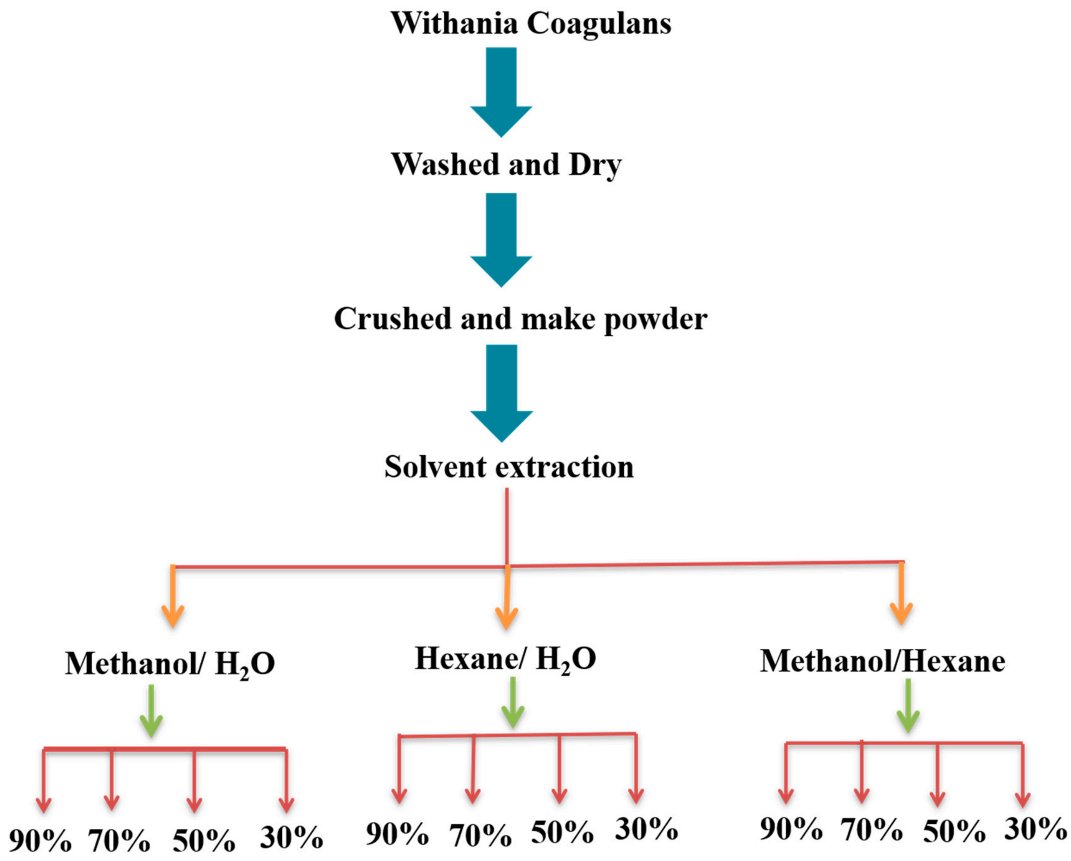

Figure. S1 Screening strategy for exploring bioactive fraction of *W. coagulans*

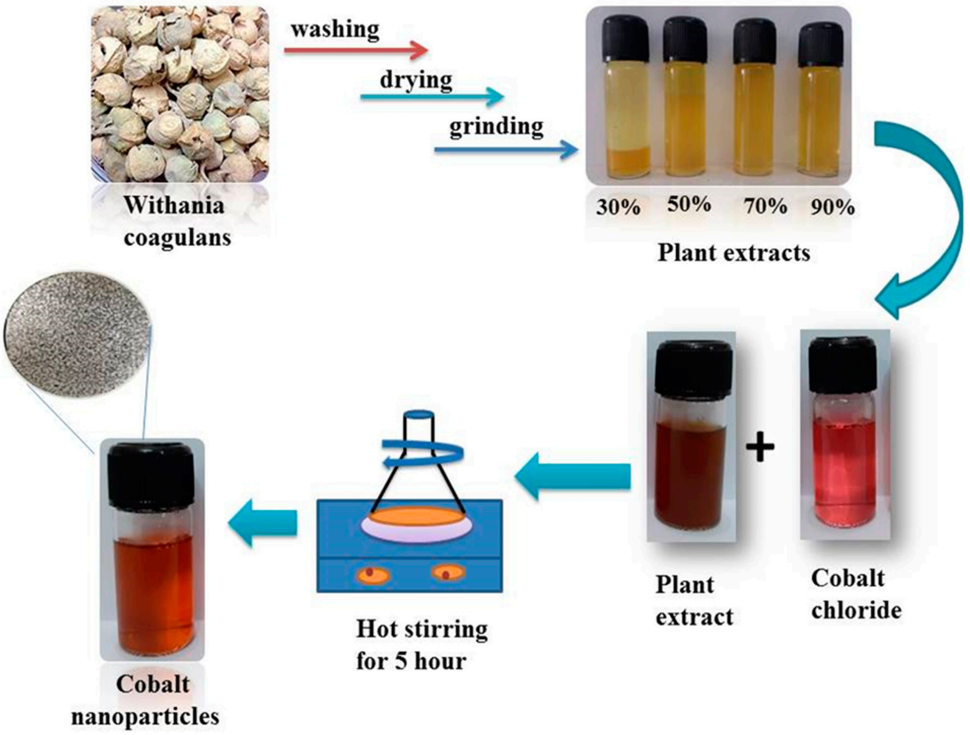

Figure. S2 Synthesis of cobalt Nanoparticles via morphological changes in reaction

Table S1 Different solvent fractionation of *Withania coagulans*

Serial #	Fractions	Code	Serial #	Fractions	Code
1	90% Methanol/H_2O	M1	7	50% Hexane/H_2O	H3
2	70% Methanol/H_2O	M2	8	30% Hexane/H_2O	H4
3	50% Methanol/H_2O	M3	9	90% Methanol/Hexane	X1
4	30% Methanol/H_2O	M4	10	70% Methanol/Hexane	X2
5	90% Hexane/H_2O	H1	11	50% Methanol/Hexane	X3
6	70% Hexane/H_2O	H2	12	30% Methanol/Hexane	X4

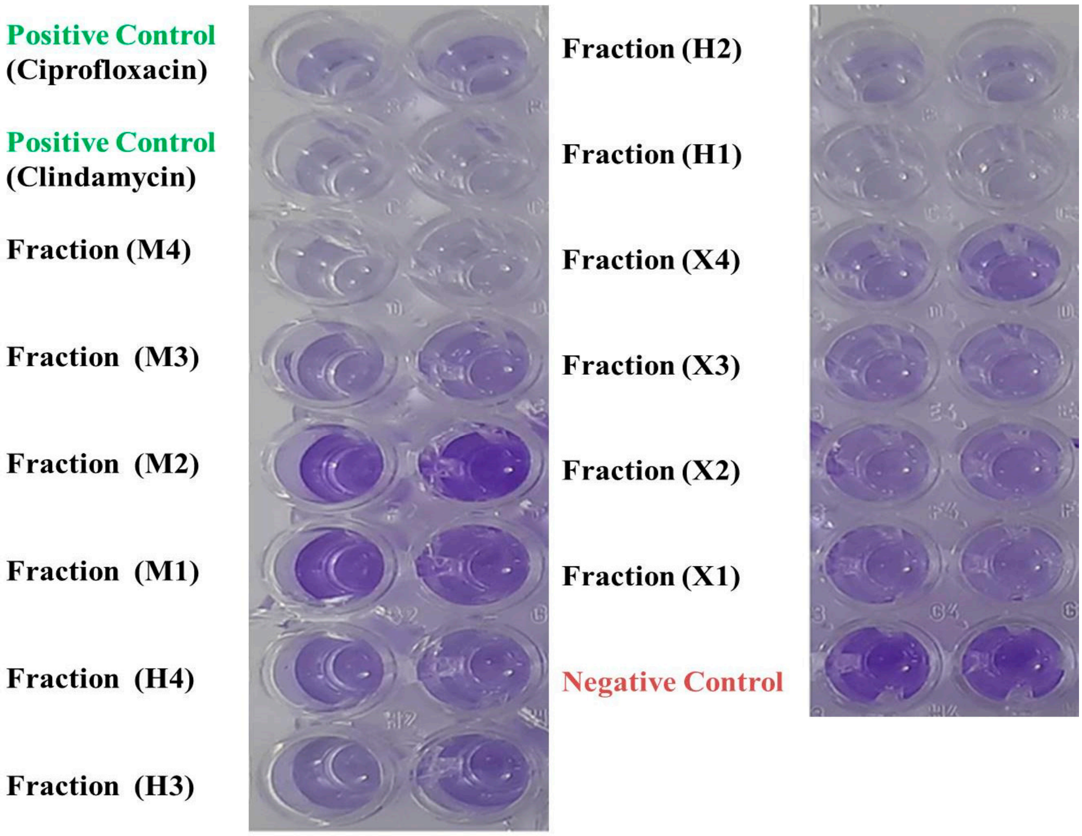

Figure S3. Anti-biofilm assay of different fractions at concentration 5mg/mL against *pseudomonas aeruginosa*

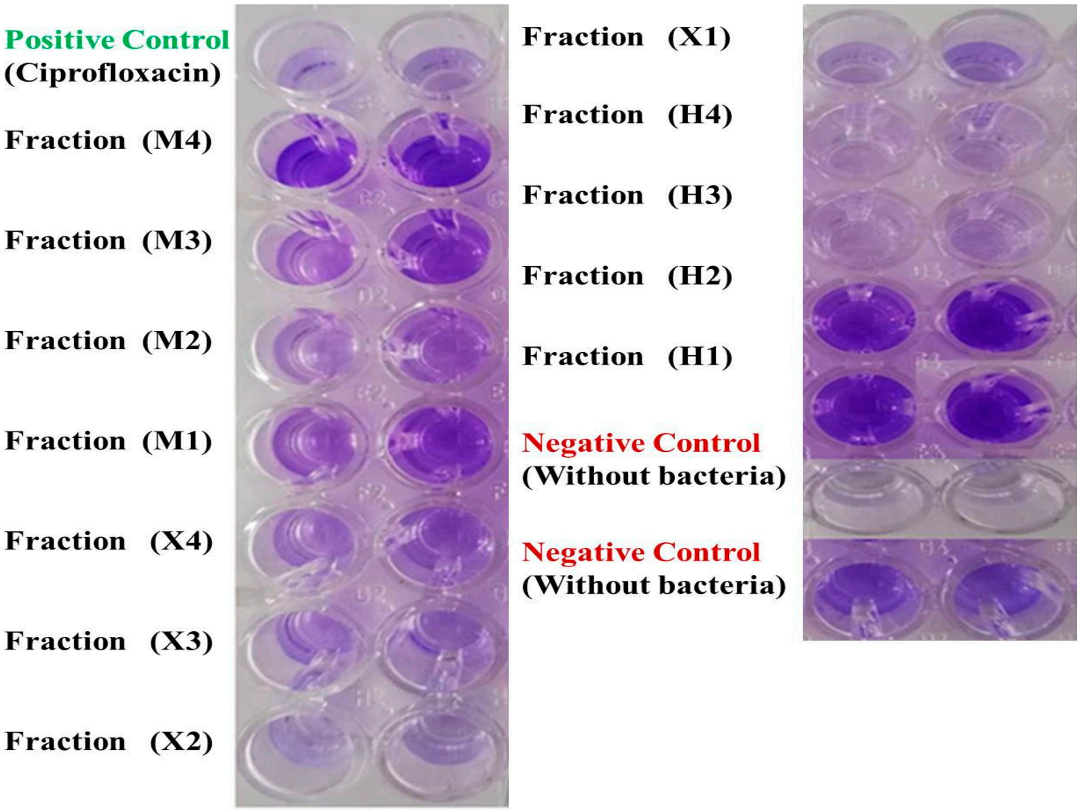

Figure S4 Anti-biofilm assay of different fractions at concentration 10mg/ml against *pseudomonas aeruginosa*

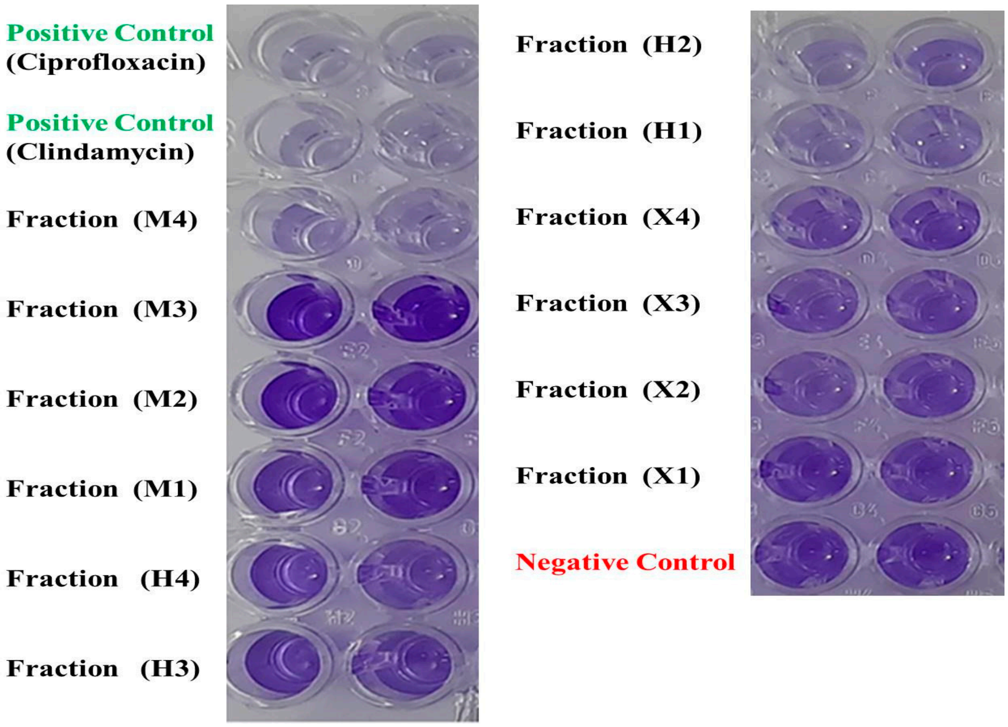

Figure S 5. Anti-biofilm assay of different fractions at concentration 5mg/ml against *Staphylococcus aureus*

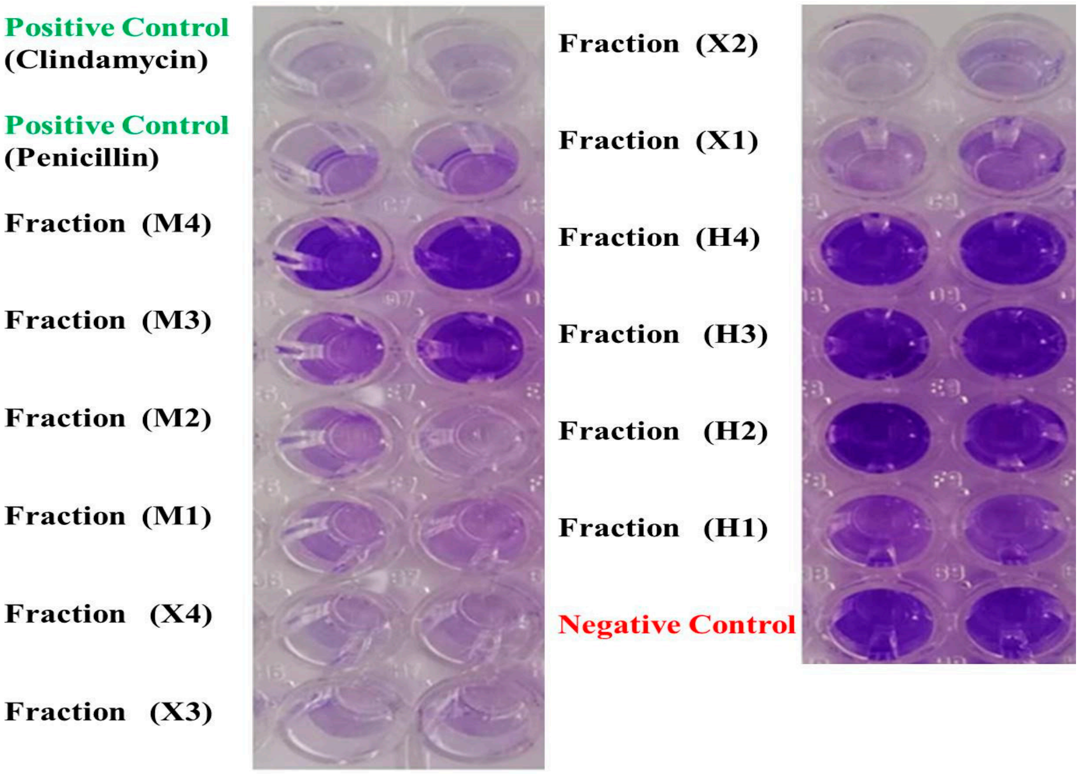

Figure S6. Anti-biofilm assay of different fractions at concentration 10mg/ml against *staphylococcus aureus*
